# Abdominal aortic aneurysm screening program in Poland

**DOI:** 10.1007/s00772-014-1348-4

**Published:** 2014-10-19

**Authors:** A. Jawien, B. Formankiewicz, T. Derezinski, A. Migdalski, P. Brazis, L. Woda

**Affiliations:** Department of Vascular Surgery and Angiology, University Hospital No.1, Collegium Medicum, University of Nicolai Copernicus, M.Sklodowskiej-Curie Street 9, 85-094 Bydgoszcz, Poland

**Keywords:** Rupture, Screening, Ultrasonography, Prevention, Risk factors, Ruptur, Screening, Ultraschall, Prävention, Risikofaktoren

## Abstract

**Background:**

Screening for abdominal aortic aneurysms (AAA) is currently recommended by several vascular societies. In countries where it has been introduced the prevalence of AAAs differed greatly and was mainly related to cigarette smoking. The screening program also had an enormous impact on the decrease of AAA ruptures and reduced mortality rate. These facts have led to the introduction of the first screening program for AAAs in Poland.

**Objective:**

The aim of the study was to determine the prevalence of AAAs among men aged 60 years and older undergoing ultrasound examination of the abdominal aorta.

**Material and methods:**

A single ultrasonography of the abdomen was performed to assess the aorta from the renal arteries to the bifurcation and the diameter of the aorta was measured at its widest point. The cut-off value for determining an aortic aneurysm was set at a diameter of ≥ 30 mm. All ultrasonography measurements were performed by physicians in outpatient departments throughout the Kuyavian-Pomeranian Province. Additionally, each subject had to fill out a questionnaire with demographic data, smoking habits, existing comorbidities and familial occurrence of AAAs. The study was conducted from October 2009 to November 2011.

**Results:**

The abdominal aorta ultrasound examinations were carried out in 1556 men aged 60 years and older. The prevalence of AAA in the study population was 6.0 % (94 out of 1556). The average age of the men was 69 years (SD 6 years, range 60–92 years). In the study population 55 % of the men smoked or had smoked and 3 % were aware of the presence of AAAs in family members. There were three risk factors significantly associated with the presence of AAAs: age (p < 0.05), smoking (72.3 % vs 53.9 %, p = 0.004) and family history of AAAs (9.6 % vs 2.7 %, p = 0.017).

**Conclusion:**

The prevalence of AAAs among men in Poland is higher than in other European countries and the USA. The screening program for AAAs is an easy and reliable method for detecting early stages of the disease and risk factors which are the driving forces for the development of AAAs.

## Abstract (de)

**Hintergrund:**

Von mehreren gefäßmedizinischen Fachgesellschaften werden derzeit Screeningprogramme für ein abdominales Aortenaneurysma (AAA) empfohlen. In den Ländern, in denen sie schon implementiert wurden, zeigten sich erhebliche Prävalenzunterschiede, AAA wurden v. a. mit Zigarettenrauchen in Verbindung gebracht. Ferner hatte das Screeningprogramm enormen Einfluss auf sinkende Rupturraten und geringere Mortalitätsraten. Diese Tatsachen haben zur Einführung des ersten Screeningprogramms für AAA in Polen geführt.

**Ziel:**

Ziel der Studie war die Bestimmung der AAA-Prävalenz bei Männern im Alter von ≥60 Jahren mittels einer sonographischen Untersuchung der Aorta abdominalis.

**Material und Methoden:**

Zur Untersuchung der Aorta zwischen Aa. renales und Bifurkation sowie zur Bestimmung des größten aortalen Durchmessers wurde eine einzelne abdominale Ultraschalluntersuchung durchgeführt. Der Cut-off-Wert zur Bestimmung eines AAA wurde bei einem Durchmesser von ≥ 30 mm gesetzt. Alle sonographischen Messungen erfolgten durch Ärzte in Ambulanzen der gesamten Provinz Kujawsko-Pomorskie. Zusätzlich war von jedem Probanden ein Fragebogen zu demographischen Details, Rauchgewohnheiten, Komorbidität und AAA-bezogener Familienanamnese auszufüllen. Durchgeführt wurde die Studie von 10/2009 bis 11/2011.

**Ergebnisse:**

Ultraschalluntersuchungen der Aorta abdominalis wurden bei 1556 Männern im Alter von ≥60 Jahren durchgeführt. Die AAA-Prävalenz im Studienkollektiv lag bei 6 % (94 von 1556), das Durchschnittsalter betrug 69 Jahre (60−92, Standardabweichung 6 Jahre). Zum Zeitpunkt der Studie oder früher waren 55 % des Kollektivs Raucher, eine AAA bei Familienmitgliedern war in 3 % der Fälle bekannt. Drei Risikofaktoren waren statistisch signifikant assoziiert mit einem AAA: Alter (p < 0,05), Nikotinkonsum (72,3 vs. 53,9 %, p = 0,004) und eine für AAA positive Familienanamnese (9,6 vs. 2,7 %, p = 0,017).

**Schlussfolgerung:**

Die AAA-Prävalenz unter in Polen lebenden Männern ist höher als die Prävalenz bei Männern anderer europäischer Länder und aus den USA. Das AAA-Screeningprogramm ist eine einfache, verlässliche Methode zur Detektion sowohl früher Stadien der Erkrankung als auch der für die Entwicklung eines AAA wesentlichen Risikofaktoren.

An abdominal aortic aneurysm (AAA) is defined as a widening of the aorta below the renal arteries with a diameter larger than 50 % of the normal measurement or with a diameter of 3 cm or more [1, 2]. The prevalence of AAAs is estimated to be 4–7 % in men aged 65 years and older [2, 3] and only 1.3 % in women of the same age [4]. Most AAAs are asymptomatic and are very often diagnosed incidentally during imaging studies performed for other diseases. The rupture of AAAs can also be the first evidence of an imminently life-threatening AAA. Mortality rates associated with rupture of an AAA remain consistently high (60–80 %, [5]). Approximately 50 % of patients die before treatment and later half of those who survived the operation can die in the perioperative and postoperative period. In contrast, elective surgery carries a much lower risk of death (3–6 %); therefore, this is why there was a need to find a way to diagnose the patients with AAA earlier and submit them to elective surgery for lowering the rates of AAA rupture and patient mortality. A single screening for AAAs by means of ultrasound examination of the aorta in men over the age of 65 years is currently highly recommended by several vascular societies. Such screening programs are now officially available in the USA, UK and Sweden [6, 7, 8].

In Poland there is so far no national screening for AAA; therefore, it was decided to introduce the first attempt of a screening program for men in one of the Polish provinces of over 2 million inhabitants (Fig. 1).

**Fig. 1:**
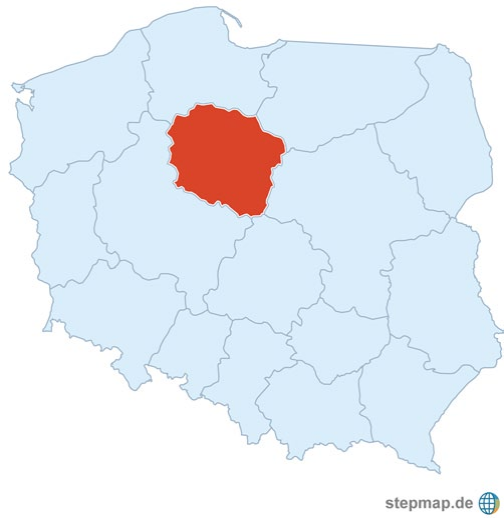
Map of Poland (38 million inhabitants) showing the location of the Kuyavian–Pomeranian Province (2 million inhabitants)

## Aim

The aim of the study was to determine the prevalence of AAA among men aged 60 years and older, undergoing ultrasound examination of the abdominal aorta. Additionally, the following were assessed: (1) relationship of selected risk factors with the presence of AAA, (2) prevalence of AAA in men at the age of 65 years and (3) prevalence of AAA in men aged 65–75 years who have ever smoked. Tasks 2 and 3 were taken into consideration because of particular differences between the UK and the USA screening programs for AAA. The assignment to such groups of screened men made it possible to compare the data with those available from the UK and the USA.

## Material and methods

The population of men aged 60 years and older was screened by single ultrasonography of the abdomen. The aorta was examined from the renal arteries to the bifurcation and the diameter of the aorta was measured in its largest point. The cut-off value for differentiating between a normal aorta and an aneurysm of the aorta was set at a diameter of ≥ 30 mm. All ultrasonography measurements were performed by physicians in outpatient practices across the Kuyavian-Pomeranian Province. Additionally, each subject had to fill out the questionnaire with demographic data, smoking habits, existing comorbidities and familial occurrence of AAA. The study was conducted from October 2009 to November 2011. Statistical analysis of the data was performed using the software STATISTICA 9 (StatSoft 200, Krakow, Poland). A p-value ≤ 0.005 was considered as statistically significant.

## Results

Over the period of 3 years the abdominal aortic ultrasound examinations were carried out in 1556 men aged 60 years and older. The prevalence of AAA in the study population was 6.0 % (94 out of 1556; Tab. 1). The average age of the men was 69 years (SD 6 years, range 60–92 years). In the study population 55 % of the men smoked or had ever smoked and 3 % were aware of the presence of AAA in family members. Of the AAAs detected by the screening program 75 % were small (less than 44 mm) and observational follow-up of ultrasound was required once a year. The AAAs detected with a diameter between 45 mm and 54 mm were observed in 13 patients (14 % of AAAs) and AAAs with a diameter equal to or exceeding 55 mm were diagnosed in 10 patients (11 %; Tab. 2).

**Tab. 1:** Prevalence of abdominal aortic aneurysms *(AAA)* in each year of performing screening

Year of recruitment	Number of screened men (n)	AAA (n)	AAA (%)
2009	496	37	7.5
2010	563	25	4.4
2011	497	32	6.4
Total	1556	94	6.0

**Tab. 2:** Distribution of the identified abdominal aortic aneurysms *(AAA)* divided into groups according to maximum diameter

AAA maximum diameter (mm)	Patients with AAA (n)	AAA (%)
30–44	71	75
45–54	13	14
55 and more	10	11
Total	94	100

Risk factors associated with AAA were analyzed and the study sample could be divided into two groups: group I men with AAA (n = 94) and group II men with a normal aorta (n = 1462). There were three risk factors significantly associated with the presence of AAA: age (p < 0.05), smoking (72.3 % versus 53.9 %, p = 0.004) and family history of AAA (9.6 % versus 2.7 %, p = 0.017; Tab. 3).

**Tab. 3:** Logistic regression analysis of risk factors associated with the presence of abdominal aortic aneurysm *(AAA)*

Risk factor	AAA n = 94	Normal aorta n = 1462	P-value	Odds ratio	95 % Confidence interval
Age (years ± SD)	72.1 ± 6.6	68.9 ± 5.9	0.05	1.08	1.04–1.11
Smoking (%)	68 (72.3 %)	789 (53.9 %)	0.004	2.02	1.26–3.25
Family history (%)	9 (9.6 %)	40 (2.7 %)	0.017	2.60	1.18–5.68

The additional task was undertaken to determine the prevalence of AAA in patients who met the criteria for inclusion of AAA screening programs in the UK and the USA. In the UK the AAA screening program includes only men aged 65 years. The number of Polish men screened at the age of 65 years was 224 during the 3 year study and AAAs were found in 9 which accounts for a prevalence of 4 %. This percentage is equal to that of the National Health Service Abdominal Aortic Aneurysm Screening Program in the UK [9]. In contrast, in the USA a single ultrasound examination of the aorta is offered to men between 65 and 75 years of age who have ever smoked. In the Polish population of men screened by using US criteria, a group of 41 AAAs out of 555 men was identified which accounts for a prevalence of 7.4 %. This percentage is higher than that of the Screen for Abdominal Aortic Aneurysms Very Efficiently (SAAAVE) Act (5.1 %) in the USA [10].

## Discussion

According to the recent European Society for Vascular Surgery (ESVS) guidelines, population screening studies offer the best evidence regarding the prevalence of AAA. This evidence is mainly based on four randomized trials which showed the reduced risk of AAA rupture in men when ultrasound screening was performed [2]. The prevalence of AAAs differs from country to country and depends on the type of screening implemented and magnitude of risk factors, such as smoking present in the screened population. The best organized screening program for AAA is currently run in the UK. It has recently been published that the propensity of the prevalence rate of AAA in the UK is on the decrease from the previous rate of 4 % to the current 1.7 % [11]. Very similar data were also obtained from the screening program in Sweden [12].

In a country like Poland, where the smoking rate among men is still very high and diet is not always properly considered, the higher prevalence of AAA should not be a surprise. The overall results with a 6 % prevalence rate of AAA correspond well with the previous UK data published in the 1990s and American data; however, screening programs for AAA in southern European countries (e.g. Italy, Spain and Portugal) have demonstrated a very similar rate of prevalence of AAA in men despite a Mediterranean diet. This has led the European guidelines to the statement that the screening of older men for AAA should be recommended in regions where the prevalence in the population is 4 % or higher because this will reduce aneurysm-related mortality by almost half within 4 years of screening, principally by reducing the incidence of aneurysm rupture [2]. The final effect of this preliminary study was the introduction in 2011 of the Kuyavian-Pomeranian AAA screening program [13]. This program is on-going and criteria for AAA screening, accepted by local authorities are similar to those of the UK, with males of age 65 years who have ever smoked. The preliminary results of last 2 years of this program (2012–2013) revealed the prevalence rate of AAA of 5.3 %. The national program of AAA screening is going to be launched in Poland in 2015. The final inclusion criteria are still to be discussed.

## Conclusion

- The prevalence of AAA among men in Poland is higher than in other European countries and the USA. A possible explanation for this can be found in the high rate of smoking and very fatty diet in the Polish population.
- To organize a well-defined screening program for AAA requires a lot of effort and very good relations with general practitioners who have access to demographic and medical records of people living in the community.
- The use of media for advertising the AAA screening program has an enormous impact on the compliance rate.
